# Cloning and sequence analysis demonstrate the chromate reduction ability of a novel chromate reductase gene from *Serratia* sp

**DOI:** 10.3892/etm.2014.2148

**Published:** 2014-12-18

**Authors:** PENG DENG, XIAOQING TAN, YING WU, QUNHUA BAI, YAN JIA, HONG XIAO

**Affiliations:** 1 Department of Occupational and Environmental Health, College of Public Health and Administration, Chongqing Medical University, Chongqing 400016, P.R. China; 2 Physical Examination Center, Third People’s Hospital of Chongqing, Chongqing 401121, P.R. China

**Keywords:** chromate reductase, flavin mononucleotide reductase, gene cloning, *Serratia* sp., sequence analysis

## Abstract

The ChrT gene encodes a chromate reductase enzyme which catalyzes the reduction of Cr(VI). The chromate reductase is also known as flavin mononucleotide (FMN) reductase (FMN_red). The aim of the present study was to clone the full-length ChrT DNA from *Serratia* sp. CQMUS2 and analyze the deduced amino acid sequence and three-dimensional structure. The putative ChrT gene fragment of *Serratia* sp. CQMUS2 was isolated by polymerase chain reaction (PCR), according to the known FMN_red gene sequence from *Serratia* sp. AS13. The flanking sequences of the ChrT gene were obtained by high efficiency TAIL-PCR, while the full-length gene of ChrT was cloned in *Escherichia coli* for subsequent sequencing. The nucleotide sequence of ChrT was submitted onto GenBank under the accession number, KF211434. Sequence analysis of the gene and amino acids was conducted using the Basic Local Alignment Search Tool, and open reading frame (ORF) analysis was performed using ORF Finder software. The ChrT gene was found to be an ORF of 567 bp that encodes a 188-amino acid enzyme with a calculated molecular weight of 20.4 kDa. In addition, the ChrT protein was hypothesized to be an NADPH-dependent FMN_red and a member of the flavodoxin-2 superfamily. The amino acid sequence of ChrT showed high sequence similarity to the FMN reductase genes of *Klebsiella pneumonia* and *Raoultella ornithinolytica*, which belong to the flavodoxin-2 superfamily. Furthermore, ChrT was shown to have a 85.6% similarity to the three-dimensional structure of *Escherichia coli* ChrR, sharing four common enzyme active sites for chromate reduction. Therefore, ChrT gene cloning and protein structure determination demonstrated the ability of the gene for chromate reduction. The results of the present study provide a basis for further studies on ChrT gene expression and protein function.

## Introduction

Chromium is widely used as an important industrial material in leather tanning, dyeing, electroplating, pigment manufacturing and other industries. However, the uncontrolled release of industrial waste has contaminated soil water systems (1). As a result of the secondary pollution and high cost, traditional, physical and chemical technologies cannot be extensively used to remedy the contaminated arable land and water. Thus, a number of microbial approaches to remedy chromium contamination have been investigated in attempts to overcome the problems (2–4).

Chromium exists in nature as two main oxidation states, hexavalent [Cr(VI)] and trivalent chromium [Cr(III)]. Cr(VI) is considered to be a more toxic form, while Cr(III) is relatively innocuous. Microbial bioremediation of chromium contamination is achieved mainly through two routes (5). One is the efflux of chromate ions from the cell cytoplasm, and the other is the direct reduction of Cr(VI) to Cr(III) by the NAD(P)H-dependent flavin mononucleotide (FMN) reductase (FMN_red) (6). Various bacteria and associated genes have been identified to reduce Cr(VI) to Cr(III) (7–9). Previous studies have demonstrated that the ChrR enzyme from *Pseudomonas putida* (10), the YieF protein from *Escherichia coli (E. coli)* and the FMN_red from *Pseudomonas aeruginosa* (PAO1) have the ability to reduce Cr(VI) to Cr(III) (11,12), all of which are members of the FMN_red protein family.

In the present study, a bacterium strain, *Serratia* sp*.* CQMUS2, that possesses high chromate resistance and rapid chromate reduction ability, was isolated from chromate-containing waste water generated in our previous research (13). The full-length DNA of ChrT from *Serratia* sp. CQMUS2 was cloned and the deduced amino acid sequence and three-dimensional (3D) structure were analyzed. The results may provide a basis for further studies on ChrT gene expression and protein function.

## Materials and methods

### Strains, media and plasmid. Serratia *sp*

CQMUS2 was subcultured in Luria-Bertani medium (Sangon Biotech Co., Ltd., Shanghai, China). *E. coli* DH5α was used as a host for gene cloning (Tiangen Biotech Co., Ltd., Beijing, China) and the pMD19-T plasmid was used as a cloning vector (Takara Bio, Inc., Otsu, Japan).

### Polymerase chain reaction (PCR)

Genomic DNA was isolated according to boiled template method (14). Following culture for 3 h, the cells of *Serratia* sp. CQMUS2 were harvested by centrifugation at 6,000 × g for 10 min. Cell pellets were washed three times with 0.01 M phosphate-buffered saline, suspended in sterile water and boiled for 10 min. Following centrifugation at 12,000 × g for 10 min, the supernatant was collected for PCR. The FMN_red gene from *Serratia* sp. AS13 *(*GenBank accession no. NC_017573) was selected as the reference sequence. A pair of primers, Scfmn1-forward (F) and Scfmn1-reverse (R), were designed and used to amplify the fragment of the *Serratia* sp. CQMUS2. The ChrT gene was amplified using 2xPfu PCR MasterMix (Tiangen Biotech Co., Ltd.), according to the manufacturer’s instructions. The PCR program was as follows: One cycle at 94°C for 3 min; 30 cycles at 94°C for 30 sec, 55°C for 30 sec and 72°C for 1 min; and one cycle at 72°C for 5 min. The primers are shown in Table I (Sangon Biotech Co., Ltd.).

Based on the acquired DNA fragment sequence, a high efficiency TAIL-PCR (hiTAIL-PCR) method was used to obtain 5′- and 3′-flanking sequences. This method required three consecutive rounds of PCR. In the first round, the Scfmn-SP1 and Scfmn-AD1 primers were used for rapid amplification of the 5′-DNA end. The second round of PCR used the product of the first round as the template, and used Scfmn-SP2 and Scfmn-AC1 as the primers. The third round of PCR used the product of the second round as the template, and used Scfmn-SP3 and Scfmn-AC1 as primers. The amplification of the 3′-end DNA fragment was the same as the 5′-end. The detailed steps and PCR parameters were performed, as described previously (15). All amplified products were run in 2% agarose gel and subsequently underwent Sanger sequencing.

The full-length ChrT gene was obtained using specific primers (Scfmn-F and Scfmn-R) corresponding to the 5′- and 3′-ends of the ChrT gene. The PCR parameters were as follows: One cycle at 94°C for 3 min; 30 cycles at 94°C for 30 sec, 56°C for 30 sec and 72°C for 1 min; and one cycle at 72°C for 5 min. The purified PCR product was cloned into a pMD19-T vector for sequencing.

### Nucleotide sequence accession number

The nucleotide sequence of ChrT was submitted onto GenBank under the accession number, KF211434.

### Sequence analysis

Sequence analysis of the gene and amino acids was conducted using the Basic Local Alignment Search Tool (http://www.ncbi.nlm.nih.gov/BLAST/). Open reading frame (ORF) analysis was performed using ORF Finder (http://www.ncbi.nlm.nih.gov/projects/gorf/). In addition, the physicochemical characteristics of the ChrT gene were analyzed using ProtParam software (http://www.expasy.ch/tools/protparam.html), while the conserved structural domain of ChrT was calculated via the Conserved Domain Database (CDD) in the National Center for Biotechnology Information (NCBI; http://www.ncbi.nlm.nih.gov/Structure/cdd/wrpsb.cgi). Multiple alignments between the ChrT gene and other bacterial FMN_red genes were conducted using Clustal X version 2.0 (16). A phylogenetic tree of FMN_red was constructed using the neighbor-joining method with Molecular Evolutionary Genetics Analysis (MEGA) software (version 4.0.2) (17). The secondary and 3D structures of ChrT were predicted using the NPS@ service (http://npsa-pbil.ibcp.fr/) and SWISS-MODEL program (http://swissmodel.expasy.org/).

## Results

### ChrT gene cloning from Serratia sp. CQMUS2

**Figure 1:**
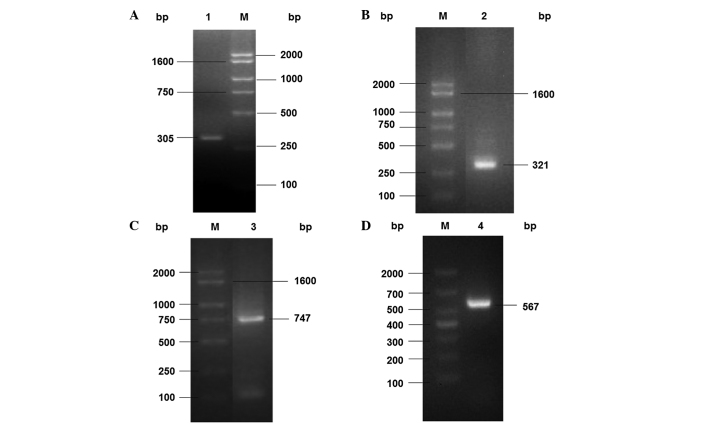
Cloning of *Serratia* sp. CQMUS2 ChrT gene fragments. (A) PCR amplification of the ChrT gene fragment: Lane M, DNA marker; lane 1, a 305-bp fragment obtained by PCR amplification with the Scfmn1-F and Scfmn1-R primers. (B) PCR amplification of the 5′-end DNA fragment: Lane M, DNA marker; lane 2, a 321-bp fragment obtained by the third round hiTAIL-PCR amplification with the Scfmn1-SP3 and Scfmn1-AC1 primers. (C) PCR amplification of the 3′-end DNA fragment: Lane M, DNA marker; lane 3, a 747-bp fragment obtained by the third round hiTAIL-PCR amplification with the Scfmn1-SP3′ and Scfmn1-AC1′ primers. (D) PCR amplification of the complete DNA: Lane M, DNA marker; lane 4, the 567-bp ChrT gene obtained by PCR amplification with the Scfmn-F and Scfmn-R primers. PCR, polymerase chain reaction; F, forward; R, reverse.

To obtain the full-length ChrT gene from *Serratia* sp. CQMUS2, a hiTAIL-PCR method was used. Firstly, a pair of specific primers was synthesized on the basis of the FMN_red gene sequence of *Serratia* sp. AS13. When the genomic DNA from the *Serratia* sp. CQMUS2 cells was used as a template, an expected 305-bp fragment of the ChrT gene was amplified using the Scfmn1-F/R primers, which was subsequently sequenced (Fig. 1A). Based on this DNA fragment, the 5′- and 3′-end fragments were amplified by 5′- and 3′-hiTAIL-PCR. Two DNA fragments were obtained in the third round of the hiTAIL-PCR. The fragments were 321 and 747 bp in length, respectively (Fig. 1B and C). Finally, on the basis of the three DNA fragment assembly, a full-length ChrT gene of 567 bp was obtained with the specific Scfmn-F/R primers (Fig. 1D). This result demonstrated that the ChrT gene was able to be successfully cloned from *Serratia* sp. CQMUS2.

### ChrT protein may be an NADPH-dependent FMN_red and a member of the flavodoxin-2 superfamily

To understand the characteristics of the ChrT protein, its nucleotide sequence was investigated. Nucleotide sequence analysis indicated that the *Serratia* sp. CQMUS2 ChrT gene contained 567 bp nucleotides with an ORF of 567 bp, which encoded a 188-amino acid peptide with a theoretical molecular weight of 20.4 kDa and an isoelectric point of 5.18. The conserved domain was predicted by CDD in NCBI, and revealed that the putative polypeptide from *Serratia* sp. CQMUS2 contained an integrated conserved region of FMN_red formed by 5–150 amino acids. Consequently, the result predicted that the deduced protein was an NADPH-dependent FMN_red and a member of the flavodoxin-2 superfamily; thus, the peptide was named as FMN_red ChrT.

### ChrT is closely associated with FMN_red members in Klebsiella pneumonia (YP_002241302), Raoultella ornithinolytica (YP_007875969) and Klebsiella oxytoca (YP_005017294.1)

**Figure 2:**
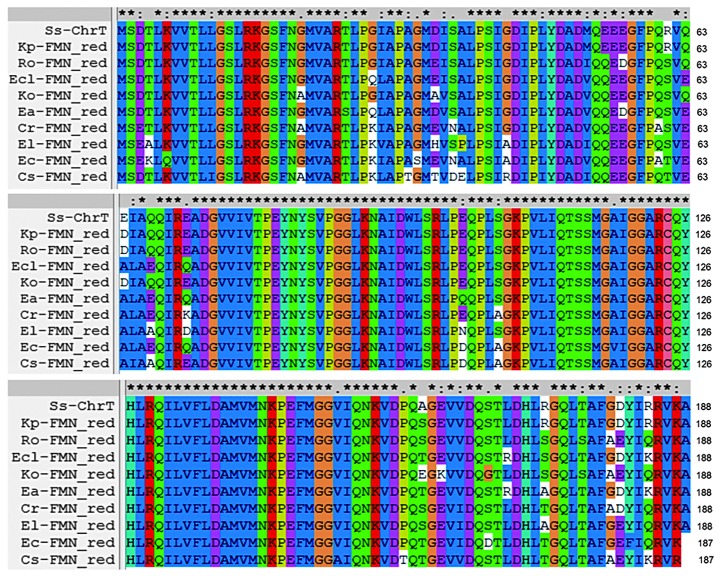
Multiple sequence alignments of the predicted protein of ChrT with FMN_red genes from other species. Ss-ChrT (*Serratia* sp. CQMUS2, KF211434); Kp-FMN_red (*Klebsiella pneumonia*, YP_002241302.1); Ro-FMN_red (*Raoultella ornithinolytica*, YP_007875969.1); Ecl-FMN_red (*Enterobacter cloacae*, YP_006479989.1); Ko-FMN_red (*Klebsiella oxytoca*, YP_005017294.1); Ea-FMN_red (*Enterobacter asburiae*, YP_004830940.1); Cr-FMN_red (*Citrobacter rodentium*, YP_003367456.1); El-FMN_red (*Enterobacter lignolyticus*, YP_003943949.1); Ec-FMN_red (*Escherichia coli*, WP_001469560.1); Cs-FMN_red (*Cronobacter sakazakii*, YP_007442551.1); FMN_red, flavin mononucleotide reductase.

**Figure 3:**
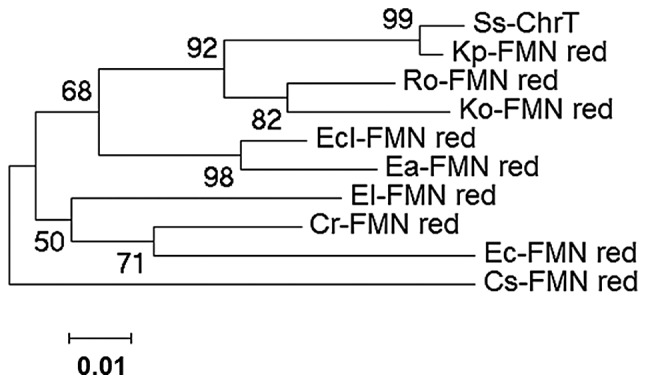
Phylogenetic trees derived from the amino acid sequences of ChrT and FMN_red genes from other species. Ss-ChrT (*Serratia* sp. CQMUS2, KF211434); Kp-FMN_red (*Klebsiella pneumonia*, YP_002241302.1); Ro-FMN_red (*Raoultella ornithinolytica*, YP_007875969.1); Ecl-FMN_red (*Enterobacter cloacae*, YP_006479989.1); Ko-FMN_red (*Klebsiella oxytoca*, YP_005017294.1); Ea-FMN_red (*Enterobacter asburiae*, YP_004830940.1); Cr-FMN_red (*Citrobacter rodentium*, YP_003367456.1); El-FMN_red (*Enterobacter lignolyticus*, YP_003943949.1); Ec-FMN_red (*Escherichia coli*, WP_001469560.1); Cs-FMN_red (*Cronobacter sakazakii*, YP_007442551.1); FMN_red, flavin mononucleotide reductase.

To compare the sequence differences between ChrT and other FMN_red members, multiple sequence alignment and phylogenetic analyses were performed. The deduced amino acid homology alignment of ChrT with the other nine FMN_red genes from different species was conducted by Clustal X software (version 2.0). As shown in Fig. 2, the sequences and GenBank accession numbers are as follows: *Klebsiella pneumonia* (YP_002241302), *Raoultella ornithinolytica* (YP_007875969), *Enterobacter cloacae* (YP_006479989.1), *Klebsiella oxytoca* (YP_005017294.1), *Enterobacter asburiae* (YP_004830940.1), *Citrobacter rodentium* (YP_003367456.1), *Enterobacter lignolyticus* (YP_003943949.1), *E. coli* (WP_001513430.1) and *Cronobacter sakazakii* (YP_007442551.1). The similarities between *Serratia* sp. CQMUS2 and the aforementioned nine bacteria were 99, 94, 92, 92, 90, 89, 88, 85 and 86%, respectively. A phylogenetic tree of the FMN_red members, including ChrT, was constructed using the neighbor-joining method with MEGA 4.0.2 software. According to the phylogenetic tree (Fig. 3), ChrT was found to be closely associated with FMN_red members, including *Klebsiella pneumonia* (YP_002241302), *Raoultella ornithinolytica* (YP_007875969) and *Klebsiella oxytoca* (YP_005017294.1).

### ChrT protein structure confers its chromate reduction ability

**Figure 4:**
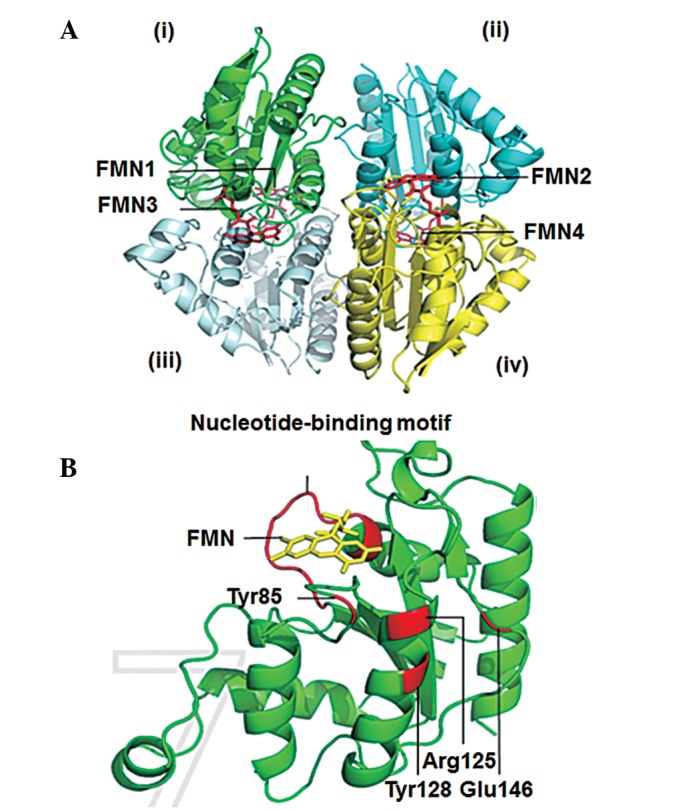
Three-dimensional structure model of ChrT predicted using the SWISS-MODEL program, according to the crystal structure of the ChrR enzyme from *E. coli*. (A) Crystal structure of the *Serratia* sp*.* CQMUS2 ChrT tetramer, which is formed by two dimers, with each monomer containing one binding site for FMN. One dimer is formed by monomers (i) and (ii), while the other dimer is formed by monomers (iii) and (iv). (B) Crystal structure of the nucleotide-binding motif showing FMN, as well as the Glu146, Tyr8, Arg125 and Tyr128 catalytic residues in ChrT. *E. coli, Escherichia coli*; FMN, flavin mononucleotide.

To predict the secondary and 3D structures of ChrT, the NPS@ service method and SWISS-MODEL program based on a known crystal structure of the *E. coli* ChrR enzyme were used, respectively. The ChrT protein was shown to contain the following structures: 40.96% α-helix, 11.70% extended strand and 47.34% random coil, but no π-helix, 3_10_-helix or other secondary structures. The predicted 3D model and template showed a high similarity of 85.6%. The structure of the predicted ChrT protein was a tetramer, formed by two symmetry-related dimers. In addition, each monomer of the dimers shows an enzyme combined with FMN that contains the enzyme-active site (Fig. 4A). Furthermore, each monomer was shown to contain the nucleotide-binding motif, GSLRKGSFN, which anchors FMN firmly, and four amino acids (Tyr128, Glu146, Arg125 and Tyr85) that are associated with chromate reductase activity (Fig. 4B). These observations demonstrated that the ChrT protein structure conferred an ability for chromate reduction.

## Discussion

In the present study, the chromate reductase ChrT gene was cloned from *Serratia* sp*.* CQMUS2 using three steps of PCR amplification based on different principles. Firstly, specific primers were designed according to the homologous sequence from *Serratia* sp. AS13 for the amplification of the ChrT gene fragment. Secondly, 5′- and 3′-flanking sequences of ChrT were obtained by a hiTAIL-PCR method. Thirdly, the full-length gene of ChrT was obtained using specific primers corresponding to its 5′- and 3′-ends. In previous studies, rapid amplification of the cDNA end method has been employed to obtain flanking sequences (18,19), where RNA is required as the template. The extraction, purification and reverse transcription of RNA are time-consuming and expensive. In particular, rapid amplification of 5′-cDNA end technology is often too difficult to succeed (20). However, the hiTAIL-PCR method uses DNA as the template; thus, has a number of advantages, including simple operation, low cost, high specificity and excellent repetition (21).

FMN exists widely in nature and participates in the electronic transmission from substrate to electron acceptor as a coenzyme of flavoenzyme. Therefore, FMN can reduce Cr(VI) to Cr(III) through electronic transmission from NAD(P)H to Cr(VI) (22). In the present study, the FMN_red protein was obtained from *Serratia* sp*.* CQMUS2, and using conserved domain analysis, the protein was demonstrated to belong to the flavodoxin-2 superfamily. The amino acid sequence of FMN_red from *Serratia* sp. CQMUS2 revealed 99 and 94% identity to the enzymes from *Klebsiella pneumonia* and *Raoultella ornithinolytica*, respectively. Moreover, the predicted 3D model revealed that the protein was a tetramer that was composed of four enzymes combined with FMN, which had a high similarity to the ChrR enzyme of *E. coli.* In a previous study, the crystal structure of the ChrR enzyme demonstrated that FMN was anchored by several hydrogen bonds to the nucleotide-binding motif, GSLRKGSFN, located on each monomer of the protein (23). In addition, the amino acids, Tyr128, Glu146, Arg125 and Tyr85, and associated hydrogen bond networks, were found to play a critical role in enhancing chromate reductase activity (23). In the present study, the predicted 3D structure of the FMN_red protein was found to contain the GSLRKGSFN nucleotide-binding motif and the amino acids, Tyr128, Glu146, Arg125 and Tyr85, in each monomer. Therefore, the predicted structure theoretically indicates that the proteins share a common catalytic mechanism for chromate reduction.

In conclusion, ChrT gene cloning and protein structure prediction demonstrated the ability of the gene for chromate reduction. Future studies should investigate ChrT expression in *E. coli* BL21, the enzyme activity and its application in removing chromium from waste water.

## Figures and Tables

**Table I:** Primers used in the study.

Procedure	Primers	Primer sequences (5′-3′)
ChrT gene fragment	Scfmn1-F	GATGTGCAACAAGACGAAGGT
	Scfmn1-R	GATGACTCCGCCCATAAACTC
5′-hiTAIL-PCR	Scfmn-SP1	GATGACTCCGCCCATAAACTCC
	Scfmn-SP2	AGACTCACCAGAGTCGATGGCGTTTTTCAGGC
	Scfmn-SP3	CTATCACAACCCCATCCGCCT
	Scfmn-AD1	ACGCTAGACTCACCTCVNVNNNGGAA
	Scfmn-AC1	ACGCTAGACTCACCTC
3′-hiTAIL-PCR	Scfmn-SP1′	AGGCGGATGGGGTTGTGATAG
	Scfmn-SP2′	GAACTGCACACGCCTGAAAAACGCCATCGACT
	Scfmn-SP3′	GTGCGCGCTGCCAGTATCAT
	Scfmn-AD1′	ACGATGAACTGCACTGVVNVNNNCCAA
	Scfmn-AC1′	ACGATGAACTGCACTG
Full-length ChrT gene	Scfmn-F	ATCATGTCAGATACCTTGAAAGTGG
	Scfmn-R	TGCTTTAACCCGCCGAATATA

PCR, polymerase chain reaction; F, forward; R, reverse; hiTAIL-PCR, high efficiency TAIL-PCR.
